# HIV patients stable on ART retain evidence of a high CMV load but changes to Natural Killer cell phenotypes reflect both HIV and CMV

**DOI:** 10.1186/s12981-015-0080-9

**Published:** 2015-12-09

**Authors:** Jacquita S. Affandi, Jacinta Montgomery, Silvia Lee, Patricia Price

**Affiliations:** Pathology and Laboratory Medicine, University of Western Australia, Nedlands, Australia; Department of Microbiology, Royal Perth Hospital, Perth, Australia; School of Biomedical Science, Curtin University of Technology, Bentley, Australia

**Keywords:** Antiretroviral therapy, CD57, CMV, HIV, Natural killer cells

## Abstract

**Background:**

Whilst ART corrects many effects of HIV disease, T cell populations retain features of accelerated immunological aging.

**Methods:**

Here we analyse phenotypic changes to natural killer (NK) cells in HIV patients who began ART with <200 CD4 T-cells/µl and maintained virological control for 12–17 years, compared with CMV seropositive and seronegative healthy control donors.

**Results:**

Humoral responses to CMV antigens (lysate, gB, IE-1) remain elevated in the patients (*P* < 0.0001) despite the long duration of ART. Patient’s NK cells responded poorly to K562 cells when assessed by CD107a and IFNγ, but this could not be attributed to CMV as responses were low in CMV-seronegative controls. Moreover HIV (and not CMV) increased expression of CD57 on CD56^lo^ cells.

**Conclusions:**

Comparisons with published studies suggest that CMV accelerates age-related increases in CD57 expression but levels plateau by 60–70 years of age, so the effect of CMV disappears. In HIV patients the plateau is higher and perhaps reached sooner.

**Electronic supplementary material:**

The online version of this article (doi:10.1186/s12981-015-0080-9) contains supplementary material, which is available to authorized users.

## Background

Over 50 % of healthy individuals and 90 % of individuals living with HIV are seropositive for cytomegalovirus (CMV). Retinitis is the most common manifestation of CMV disease in human immunodeficiency virus-1 (HIV) -infected individuals, affecting up to 40 % of American acquired immune deficiency syndrome (AIDS) patients [1], and 5–25 % of HIV patients in the developing world [2]. As CMV prophylaxis is expensive, it is suspended once patients are stable on antiretroviral therapy (ART). However CMV establishes latency with frequent reactivation triggered by inflammatory mediators, including tumour necrosis factor (TNF) [3]. Immune activation persists in HIV patients responding to ART [4] and levels of TNFα remain elevated (e.g. [5]), so subclinical reactivations of CMV triggered by these factors may continue on ART [3]. Indeed titres of antibody reactive with CMV increase in the first year on ART. They decline thereafter but remain above those seen in age-matched healthy controls. This cannot be explained by hypergammaglobulinaemia [6] and likely reflects a high burden of CMV persisting on ART.

Associations between CMV replication and cardiovascular disease include studies of clinical material linking atherosclerosis with induction of adhesion molecules in endothelial tissues actively infected with CMV [7]. This fits clinical evidence associating increased T-cell responses to CMV with more severe cardiovascular changes in HIV patients [8]. In addition, CMV drives T-cells to replicative senescence. In older CMV-seropositive adults (not infected with HIV), up to 23 % of the T-cell population can be CMV-specific [9, 10]. CMV-specific T-cells carry an immunosenescent phenotype that includes expression of CD57 and limited proliferative potential. T-cell senescence can impair T-cell homeostasis when thymic activity is depressed, as it is in HIV patients. Indeed T-cell expression of CD57 and an associated switch from interleukin (IL)-2 to interferon gamma (IFNγ) production are evident in HIV patients with a stable response to ART [11].

Several lines of evidence suggest that natural killer (NK) cells are important in the control of CMV. Direct evidence is provided by a congenitally T-cell deficient child whose acute CMV infection was accompanied by an expansion of NK cells which resolved with her plasma CMV-deoxyribonucleic acid (DNA) [12]. Teleological evidence also implicates NK cells. Human and mouse CMV diverged with their host species during mammalian evolution but both encode host proteins able to subvert NK-mediated killing [13]. In HIV patients, NK cells also retain the imprint of the pre-ART immune system for many years. For example after 8 years on ART, IFNγ responses of CD4 T-cells to CMV were *inversely* related to CD4 T-cell counts before ART in patients who began ART with <60 CD4 T-cells/μL, but IFNγ responses of NK cells to a target cell lacking ligands for inhibitory receptors (K562 cells) were *directly* proportional to nadir CD4 T-cell counts [14]. NK cell IFNγ responses remained lower than in controls, confirming persistent NK dysfunction [15].

Here we address whether phenotypic profiles of NK cells in HIV patients stable on ART remain abnormal and reflect the immunological *“footprint”* of CMV. For this purpose, the lifetime burden of CMV is estimated from the levels of antibodies reactive with CMV lysate, glycoprotein B (gB) protein or immediate early (IE)-1 antigen, or CD4 T-cell IFNγ responses.

Leukocyte immunoglobulin-like receptor-1 [LIR-1 (ILT2, LIRB1, CD85j)] expression was proposed as an early marker of CMV replication in transplant recipients, as its expression is increased before CMV-DNA appeared in plasma [16, 17]. Increased expression of LIR-1 on NK cells was associated with atherosclerosis [18], consistent with a role for CMV in atherogenesis. CD57 expression by NK cells marks terminal differentiation. CD57^+^CD56^lo^NKG2C^+^ NK cells have poor proliferative capacity and are enriched in organ transplant patients with active CMV infections [19, 20]. Our study describes HIV patients who began ART with <200 CD4 T-cells/µl and maintained virological control for 12–17 years. We selected this narrow demographic band as it remains the best long-term outcome for patients beginning ART with advanced HIV disease. It is pertinent to establish the benefits that they may accrue from CMV therapy.

## Results

### Humoral responses to all CMV antigens remain elevated in HIV patients stable on ART

All HIV patients had begun ART with low CD4 T-cell counts and achieved significant immune reconstitution [median (interquartile range): nadir 42 (23–142) CD4 T-cells/µl and current 691 (576–889) CD4 T-cells/µl after 174 (165–185) months on ART]. HIV patients and CMV seropositive (CMV+) healthy controls were similar in age, but the CMV seronegative (CMV−) controls were a little younger (Table 1).

**Table 1:** HIV patients stable on ART retain high titres of CMV reactive antibodies, with NK cell phenotypes and function affected by HIV and CMV status

	HIV patients	CMV+ controls	CMV− controls	*A vs B*	*A vs C*	*B vs C*
	A	B	C	*P* ^*b*^	*P* ^*b*^	*P* ^*b*^
*n*	20	16	9			
Male:female	19:1	14:2	9:0	0.57^c^	1	0.52
Age (years)	62 (57–68)^a^	60 (57–62)	55 (53–59)	0.19	**0.04**	0.11
CMV lysate IgG (AU/ml)	94 (56–240)	20 (10–35)	0.9 (0.6–1.0)	**<0.0001**	**<0.0001**	**<0.0001**
CMV gB IgG (AU/ml)	127 (90–172)	45 (27–55)	1.9 (1.1–2.8)	**<0.0001**	**<0.0001**	**0.0001**
CMV IE–1 IgG (AU/ml)	49 (21–166)	8 (4.3–180)	2.6 (2.0–3.7)	**<0.0001**	**<0.0001**	**0.0017**
CMV lysate IFNγ^e^	227 (16–700)	157 (13–617)	0 (0–0.5)	0.16	**<0.0001**	**<0.0001**
NK cells (% lymphocytes)	12 (8.3–18)	17 (11–20)	17 (12–22)	*0.09*	*0.07*	0.76
CD8 T-cells (% lymphocytes)	39 (19–48)	17 (7–23)	13 (10–19)	**0.0007**	**0.001**	0.71
CD56^hi^/CD56^lo^ ratio × 100	7.2 (3.5–18)	7 (2.5–9.8)	12 (9.0–26)	0.51	**0.01**	*0.06*
IFNγ^+^ (% NK cells)^d^	0.2 (0.1–0.32)	0.1 (0.1–0.38)	0.4 (0.15–0.55)	0.93	0.19	0.17
IFNγ^+^ (% NK cells) + K562	0.8 (0.3–1.10)	1.7 (1.0–2.6)	1 (0.65–2.75)	**0.006**	0.21	0.4
CD56^hi^IFNγ^lo^ (% NK cells)^d^	0.36 (0.07–1.3)	1.1 (0.48–1.6)	0.4 (0.16–0.97)	*0.08*	0.96	0.12
CD107a^+^ (% NK cells)^d^	6.3 (2.6–8.6)	6.2 (4.6–7.9)	3.0 (2.2–6.0)	0.86	0.16	**0.03**
CD107a^+^ (% NK cells) + K562	11 (6.3–14)	16 (9.8–17)	8.4 (6.1–11)	**0.03**	0.48	**0.006**
LIR–1 MFI (NK cells)	554 (419–706)	524 (351–774)	301 (270–734)	0.82	0.27	0.17
LIR–1 MFI (CD8 T cells)	494 (385–679)	526 (429–852)	374 (283–807)	0.3	*0.07*	0.15
CD57^+^ (%NK cells)	77 (59–83)	64 (54–66)	63 (59–65)	**0.02**	*0.09*	0.98
CD56^lo^ perforin^hi^ (% NK cells)^d^	85 (76–90)	83 (77–89)	79 (70–87)	0.77	0.29	0.25
CD56^hi^ perforin^lo^ (% NK cells)^d^	3.0 (1.0–5.9)	3.1 (1.7–5.3)	5.2 (2.9–5.3)	0.88	*0.07*	*0.08*

^a^Median (interquartile range)

^b^Mann–Whitney tests (continuous data), *P* ≤ 0.05 (bold), *P* > 0.05–0.1 (italics)

^c^Fisher’s exact test (categorical data)

^d^Cultured without K562 cells

^e^IFNγ spots per 2 × 10^6^ cells

Levels of antibodies reactive with CMV lysate, CMV gB and CMV IE1 were higher in HIV patients than CMV+ controls. In HIV patients, levels of antibodies reactive with CMV antigens were tightly correlated (R = 0.57–0.81, *P* = 0.009 to <0.0001). The relationship was similar in CMV+ controls (R = 0.52–0.57, *P* = 0.02–0.18) and levels of antibody reactive with lysate and gB were correlated in CMV− controls (R = 0.75, *P* = 0.025), consistent with everyone having some exposure to CMV in their lifetime. Antibody levels were not influenced by age, perhaps because the age range was limited (50–73 years). Antibody levels in HIV patients were steady or declined marginally on ART (R = −0.34 to −0.05, *P* = 0.14–0.85).

Compared with the clear difference in humoral responses to CMV, CD4 T-cell IFNγ responses to CMV lysate did not differ between HIV+ CMV+ patients and CMV+ controls (*P* = 0.16). However IFNγ and antibody responses to CMV lysate were suggestive of association in CMV+ controls (R = 0.44, *P* = 0.08), but not in patients (R = 0.25, *P* = 0.29). From these comparisons, we selected antibody titres rather than IFNγ responses to assess the footprint of CMV on NK cells in HIV patients.

### NK cell function is depressed by stably treated HIV disease, without the boost to NK function attributed to CMV in controls

HIV patients had higher proportions of CD8 T-cells and slightly lower proportions of NK cells than CMV+ or CMV− controls individually (Table 1), with significantly less NK cells than the combined control groups (*P* = 0.03). Whilst the proportions of CD56^lo^ and CD56^hi^ cells did not differ between groups, the CD56^hi^/CD56^lo^ ratio was slightly higher in CMV− controls (*P* = 0.01 and *P* = 0.06 *vs* HIV patients and CMV+ control resp). These differences are not considered clinically informative.

NK cell function was then assessed from the proportion of NK cells expressing CD107a or IFNγ with and without stimulation with K562 cells. These markers were predominantly expressed on CD56^lo^ NK cells (Additional file 1: Figure S1) and their expression was invariably increased by culture with K562 cells. After stimulation with K562 cells, expression of CD107a and IFNγ by NK cells was lower in HIV patients than in CMV + controls (Table 1). Moreover NK IFNγ responses were lower in HIV patients who had been on ART for longer periods (R = −0.60, *P* = 0.01), with CD107a following a similar trend (R = −0.34, *P* = 0.15). IFNγ production gated specifically to CD56^hi^ NK cells was not increased by K562 cells in any group (*P* = 0.46–0.96; data not shown), but followed a pattern similar to that seen with CD56^lo^ NK cells. Specifically, expression in unstimulated cultures from HIV patients was slightly lower than from CMV + controls and was inversely proportional to time on ART (R = −0.41, *P* = 0.09).

CD107a responses were lowest in CMV- controls, so the low NK responses of HIV patients occur despite their greater humoral responses to CMV. Indeed HIV patients displayed variable but always *negative* correlations between CD107a expression (±K562 cells) and CMV antibody (lysate, gB, IE-1; R = −0.11 to −0.48). IFNγ production by CD56^lo^ or CD56^hi^ NK cells did not correlate consistently with levels of CMV antibody in any group of donors (R = −0.58 to 0.33).

### LIR-1 expression was not related to levels of CMV antibody, but associated with CD107a responses in HIV patients

The expression of LIR-1 on NK cells and CD8 T-cells was similar in CMV+ HIV patients and control donors, and slightly lower in CMV− controls. This trend was evident when LIR-1^+^ cells were assessed as a percentage of NK cells or CD8 T-cells (data not shown) or by the median fluorescent intensity (MFI) of LIR-1 (Table 1; Additional file 1: Figure S1), but did not reach statistical significance. Levels of antibody reactive with CMV did not correlate with the MFI of LIR-1 on NK cells or CD8 T-cells in HIV patients (R = −0.38 to 0.06) or CMV+ (R = −0.17 to 0.49) or CMV− (R = −0.40 to −0.10) controls.

LIR-1 is an inhibitory receptor but its ligand [human leukocyte antigen-G (HLA-G)] is not found on K562 cells [21], so correlations between LIR-1 expression and NK responses to this target are indirect. In HIV patients, NK expression of CD107a correlated with expression of LIR-1 on NK cells (Table 2) and CD8 T-cells (*P* = 0.05, R = 0.45 unstimulated; *P* = 0.01, R = 0.55 with K562). CD107a responses did not correlate with expression of LIR-1 on NK cells (Table 2) or CD8 T-cells (*P* > 0.10; R = 0.20–0.49) in CMV+ or CMV− controls, but this may reflect the smaller sample sizes. Correlations were also clearer in patients than CMV+ or CMV− controls when LIR-1^+^ cells were gated (data not shown).

**Table 2:** In HIV patients, NK expression of CD57 correlated directly with CD107a^+^ and perforin^hi^ expression on CD56^lo^ cells and inversely with IFNγ responses and the CD56^hi^ perforin^lo^ phenotype

*Population assessed as % NK cells expressing….*	HIV patients		CMV+ controls		CMV− Controls	
	*P* ^a^	*R* ^a^	***P***	***R***	***P***	***R***
LIR-1 (MFI on NK cells) *vs….*						
CD107a	**0.01**	**0.56**	0.15	0.38	0.49	0.26
CD107a with K562	**0.02**	**0.31**	0.26	0.31	0.23	0.45
CD57 *vs….*						
CD107a	**0.02**	**0.56**	0.73	0.09	0.43	−0.3
CD107a with K562	*0.08*	*0.40*	0.28	−0.30	0.70	0.15
IFNγ	0.10	−0.41	*0.06*	−*0.48*	0.61	−0.2
IFNγ with K562	0.14	−0.35	*0.09*	−*0.44*	0.17	0.5
CD56^lo^perforin^hi^	**0.001**	**0.72**	0.28	0.29	0.49	0.27
CD56^hi^perforin^lo^	**0.004**	**−0.64**	0.86	0.05	0.64	0.23

^a^Spearman’s correlation test , *P* < 0.05 (bold), *P* > 0.05–0.1 (italics)

### CD57 expression was not related to levels of CMV antibody, but was increased and correlated with CD107a and perforin responses of CD56^lo^ NK cells in HIV patients

CD57 was expressed on a higher proportion of NK cells from HIV patients than CMV+ controls (*P* = 0.02). Expression was similar in CMV+ and CMV− controls, and was not proportional to levels of CMV antibody in HIV patients (*P* = 0.22–0.57, R = −0.13 to −0.29). Examination of the flow cytometry plots confirmed that CD57 was expressed on CD56^lo^ NK cells in all donors (Additional file 1: Figure S1; [19]).

In HIV patients, CD57 expression on NK cells correlated directly with expression of CD107a, with a weak inverse association with IFNγ (±K562 cells). HIV patients also demonstrated a striking correlation between CD57 expression on NK cells and the CD56^lo^Perforin^hi^ phenotype. This was complemented by an inverse correlation between CD57 and the CD56^hi^Perforin^lo^ phenotype (Table 2). No associations were seen in control donors.

## Discussion

The effects of HIV disease and ART on NK phenotypes are unclear. Azzoni et al. [21] describe low NK cell numbers and poor NK cell cytotoxicity during untreated HIV infection, with limited recovery over 1–2 years on ART. Goodier et al. [22] reported that NK IFNγ responses remain low on ART despite in vitro stimulation with IL-2, IL-12 and IL-15. However Alter et al. [23] demonstrated increased IFNγ and CD107a responses per NK cell in viremic patients with resolution over 1 year on ART. The contribution of CMV to changes attributed to HIV may be critical and is addressed here.

Our study describes patients who began ART with <200 CD4 T-cells/µl and maintained virological control for 12-17 years. We confirmed that humoral responses to CMV antigens (lysate, gB, IE-1) remain elevated above levels seen in CMV-seropositive controls (*P* < 0.0001) and linked this with T-cell senescence and low nadir CD4 T-cell counts [24]. Patients had slightly lower proportions of NK cells but their ratios of CD56^hi^/CD56^lo^ cells were similar to controls.

We then assessed the functional capacity of NK cells using IFNγ and expression of CD107a by NK cells. IFNγ is produced when NK cells become activated [25], whereas CD107a lines the membrane of cytolytic granules and is detected when the granule fuses with the cell membrane [26]. HIV patients responded poorly to K562 cells when assessed by CD107a and IFNγ on CD57^lo^ NK cells, but this could not be attributed to their burden of CMV as responses were lowest in CMV-seronegative controls. In contrast, HIV patients described by Lima et al. [27] displayed normal to elevated NK responses to phorbol myristate acetate (PMA)/calcium (Ca) ionophore. This may reflect the means of stimulation, as PMA/Ca ionophore bypasses the NKG2D-mediated mechanisms invoked by K562 cells [28].

LIR-1 has been proposed as a measure of the *“footprint”* of CMV as its expression is increased on NK cells from CMV-seropositive individuals [16–18]. LIR-1 expression on NK cells has not been monitored during HIV infection but LIR-1 is implicated in the control of HIV replication [29]. Here expression of LIR-1 was marginally lower in CMV seronegative controls, but was not affected by HIV or proportional to levels of CMV antibody in any group. Whilst this precludes its use as a measure of the CMV footprint in this setting, we noted a relationship between LIR-1 and CD107a expression in HIV patients. This probably means that LIR-1 expression is a property of NK cells that are healthy enough to kill, as LIR-1 is an inhibitory receptor and is not implicated in the killing of K562 cells since they don’t express HLA-G [28].

CD57 has been proposed as a marker of “memory” NK cells that have been expanded in response to CMV, as NK cells co-expressing the activating NKG2C receptor and CD57 are expanded by CMV seropositivity in healthy college students, but not by active infection with Epstein-Barr virus (EBV) [30]. Here HIV (and not CMV) increased the expression of CD57 on CD56^lo^ cells. This was also seen in a mixed cohort of viraemic and aviraemic HIV patients (n = 13), where patients displayed 40 % CD57^+^ NK cells *vs* 20 % in controls. CD57 expression correlated with levels of CMV antibodies in each group [30], which was not evident in our study. However the patients and controls had a broad range of ages (25–66 years). A recent study [31] showed expression of CD57 increased with age from ~50 % of CD56^lo^ NK cells in CMV+ donors aged under 35 years to 65–70 % in those >70 years old. CMV-seronegative younger donors had just 30–35 % CD57^+^ NK cells. This is consistent with our data as our control donors (CMV+ and CMV−) were 50–70 years old with ~64 % CD57^+^ NK cells. It makes our finding that 77(59–83) % of NK cells from HIV patients express CD57 all the more striking. We conclude that CMV accelerates age-related increases in CD57 expression but levels plateau by 60–70 years of age. In HIV patients the plateau is higher and perhaps reached sooner.

Overall, the effects of HIV disease on NK cells in patients stably controlled on ART were most clearly marked by expression of CD57. Elevated expression of CD57 on NK cells from HIV patients is likely to have functional consequences as it correlated directly with CD107a and perforin by CD56^lo^ cells and inversely with IFNγ responses and the CD56^hi^ perforin^lo^ phenotype. In this older and stably treated patient cohort, CD57 expression was not proportional to the burden of CMV as measured by levels of antibody. The inclusion of rare CMV-seronegative HIV patients and parallel sensitive assessments of CMV DNA are needed to define the roles of CMV and HIV in NK dysfunction.

## Methods

### Patients and controls

Twenty CMV-seropositive HIV patients were recruited at Royal Perth Hospital, Western Australia, on the basis of being >50 years old with nadir CD4 T-cell counts <200 cells/µl, with >10 years on ART and >1 year of complete viral suppression (<50 HIV RNA copies/ml). ART comprised at least three antiretroviral drugs. Sixteen CMV-seropositive healthy controls (CMV+) and nine male CMV-seronegative healthy controls (CMV−) were included. All donors were Caucasian. CMV seropositivity in patients and controls was defined as >1100 AU/mL CMV lysate antibody, where the cut-off was based on eleven samples from persons negative by routine serology and detection of CMV DNA (Abbott, Lake Forrest, IL, USA). Informed consent was obtained from all participants in accordance with approvals from Royal Perth Hospital.

### Sample collection and storage

Peripheral blood mononuclear cells (PBMC) were isolated by ficoll density gradient centrifugation (Ficoll-Paque Plus; GE Healthcare Biosciences, Sweden). 2.0 × 10^6^ cells were set aside for flow cytometry, whilst remaining cells were resuspended at 10 × 10^6^ cells/mL in fetal calf serum (FCS) with 10 % dimethyl sulfoxide (DMSO) for storage in liquid nitrogen.

### CMV antibody and IFNγ responses

IgG reactive with CMV was quantitated using CMV lysate, CMV gB and CMV IE-1 antigens. CMV lysate was prepared by sonication of human foreskin fibroblasts (HFF) infected with CMV strain AD169. Uninfected HFF were prepared and extracts were run in parallel. Replicate ELISA plates were coated with CMV gB (2800-800 produced in hamster ovary cells, Chiron Diagnostics, Medfield, MA, USA; 50 ng/mL) and CMV IE-1 (130-092-137, produced in *E.coli*; Miltenyi Biotech, Bergisch Gladbach, Germany; 2500 ng/mL). Plasma samples were diluted from 1:200. Binding was detected using goat anti-human IgG conjugated horse radish peroxidase (HRP), followed by tetramethylbenzidine (TMB) substrate (Sigma-Aldrich; St Louis, MI, USA). Four-parameter logistic standard curves were generated from titrations of a plasma sample assigned a value of 1100 arbitrary units (AU) IgG reactive with each and unit values were derived for all samples. Units of antibody detected on plates coated with uninfected HFF were subtracted from those generated using CMV-coated plates. IFNγ responses of CD4 T-cells were assessed by ELISpot assay as described previously [11].

### NK cell and T cell phenotypes assessed ex vivo by flow cytometry

Fresh PBMC (0.5 × 10^6^ cell per tube) were resuspended in 20 μl phosphate buffered saline (PBS) with 1 % bovine serum albumin (BSA) and antibodies were added for 15 min (room temperature). This included PE-ILT2 (LIR-1) clone GHI/75 (Biolegend San Diego, CA, USA), APC-CD57 clone NK-1, PerCP-Cy5.5-CD3 clone SK7, V450-CD56 clone B159, V500-CD8 clone RPA T8 (BD Biosciences, San Jose, CA, USA). 250,000 events were acquired using a FACS Canto II cytometer (BD Biosciences) and analyzed with FlowJo software (Tree Star, San Carlos, CA, USA). Gating strategies are illustrated in Additional file 1: Figure S1.

### Assessment of NK function

K562 cells were propagated in RPMI/10 % FCS and sub-cultured 24 h before use. Cryopreserved PBMC were washed in RPMI and cultured in RPMI/5 % FCS at 1 × 10^6^ cells/mL with K562 cells at a target:effector ratio of 10:1. After 6 h (37 °C), cells were transferred to flow tubes in PBS/1 %BSA, washed and 10 μL FcR Block (Miltenyi Biotech) was added for 20 min (4 °C). Cells were stained for extracellular markers [PerCP-Cy5.5-CD3, V450-CD56, V500-CD8, APC-CD107a clone H4A3] (BD Biosciences) for 15 min (room temperature). They were then washed, 250 μL Cytofix/Cytoperm (BD Biosciences) was added for 20 min (4 °C), followed by antibodies reactive with intracellular markers [FITC-perforin clone γG9; PE-IFNγ clone 4S.B3] (BD Biosciences) for 30 min. Washed cells were resuspended in PBS/1 % BSA and 500,000 events were acquired and analyzed (Additional file 1: Figure S1).

### Statistical analysis

Results were presented as median (interquartile range) unless otherwise stated. Bivariate analyses were based on Mann–Whitney tests or Fisher’s test as indicated in the Table 1. Correlation coefficients were determined by the Spearman’s Rank Correlation Test (GraphPad Prism version 5 for Windows, GraphPad Software, La Jolla, CA, USA). For all tests, *P* < 0.05 was considered to represent a significant difference and highlighted in bold, whereas 0.05 < *P* < 1 is italicized.

## Additional file

10.1186/s12981-015-0080-9 The figure summarises the gating strategy used to define NK cell subpopulations, illustrated using typical control donors.

## Abbreviations

AIDS: Acquired immunodeficiency syndrome
ART: Antiretroviral therapy
AU: Arbitrary units
BSA: Bovine serum albumin
Ca: Calcium
CD: Cluster of differentiation
CMV−: CMV seronegative
CMV: Cytomegalovirus
CMV+: CMV seropositive
DMSO: Dimethyl sulfoxide
DNA: Deoxyribonucleic acid
EBV: Epstein-Barr virus
ELISA: Enzyme linked immunosorbent assay
ELISpot: Enzyme linked immunosorbent spot assay
FCS: Fetal calf serum
gB: Glycoprotein B
HFF: Human foreskin fibroblasts
HIV-1: Human immunodeficiency virus-1
HLA−: Human leukocyte antigen
HRP: Horseradish peroxidase
IE1: Immediate early 1 protein
IFNγ: Interferon gamma
IgG: Immunoglobulin G
IL-: Interleukin-
LIR-: Leukocyte immunoglobulin-like receptor-
MFI: Mean fluorescence intensity
NK: Natural killer cells
NKG2D: Natural-killer group 2, member D
PBMC: Peripheral blood mononuclear cells
PBS: Phosphate buffered saline
PMA: Phorbol myristate acetate
RNA: Ribonucleic acid
RPMI: Roswell Park Memorial Institute medium
TMB: Tetramethylbenzidine
TNF: Tumour necrosis factor
