# Supplementary figures and images for: HIV patients stable on ART retain evidence of a high CMV load but changes to Natural Killer cell phenotypes reflect both HIV and CMV
Source: AIDS Res Ther. 2015 Dec 9;12:41. doi: 10.1186/s12981-015-0080-9 (PMC4673723; doi:10.1186/s12981-015-0080-9)

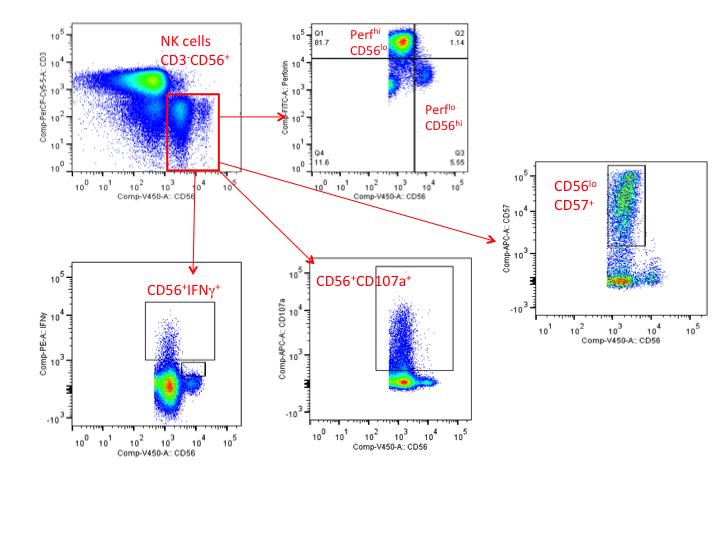

Supplement: Supplementary file 1 — 10.1186/s12981-015-0080-9 The figure summarises the gating strategy used to define NK cell subpopulations, illustrated using typical control donors. [file 12981_2015_80_MOESM1_ESM.tiff]
